# Accurate fundamental invariant-neural network representation of *ab initio* potential energy surfaces

**DOI:** 10.1093/nsr/nwad321

**Published:** 2023-12-20

**Authors:** Bina Fu, Dong H Zhang

**Affiliations:** State Key Laboratory of Molecular Reaction Dynamics and Center for Theoretical and Computational Chemistry, Dalian Institute of Chemical Physics, Chinese Academy of Sciences, Dalian 116023, China; Hefei National Laboratory, Hefei 230088, China; University of Chinese Academy of Sciences, Beijing 100049, China; State Key Laboratory of Molecular Reaction Dynamics and Center for Theoretical and Computational Chemistry, Dalian Institute of Chemical Physics, Chinese Academy of Sciences, Dalian 116023, China; Hefei National Laboratory, Hefei 230088, China; University of Chinese Academy of Sciences, Beijing 100049, China

**Keywords:** FI-NN, full-dimensional PESs, multi-channel reaction, reaction mechanism

## Abstract

Highly accurate potential energy surfaces are critically important for chemical reaction dynamics. The large number of degrees of freedom and the intricate symmetry adaption pose a big challenge to accurately representing potential energy surfaces (PESs) for polyatomic reactions. Recently, our group has made substantial progress in this direction by developing the fundamental invariant-neural network (FI-NN) approach. Here, we review these advances, demonstrating that the FI-NN approach can represent highly accurate, global, full-dimensional PESs for reactive systems with even more than 10 atoms. These multi-channel reactions typically involve many intermediates, transition states, and products. The complexity and ruggedness of this potential energy landscape present even greater challenges for full-dimensional PES representation. These PESs exhibit a high level of complexity, molecular size, and accuracy of fit. Dynamics simulations based on these PESs have unveiled intriguing and novel reaction mechanisms, providing deep insights into the intricate dynamics involved in combustion, atmospheric, and organic chemistry.

## Teaser

Substantial progress has been made in the FI-NN representation of highly accurate, global, full-dimensional PESs for multi-channel reactions with more than 10 atoms, involving many intermediates, transition states and products.

## INTRODUCTION

Chemical reactivity involves the fundamental process of breaking existing chemical bonds and forming new ones. In the case of a bimolecular reaction, when two reactant molecules collide, atoms rearrange themselves to generate new product molecules. The comprehensive understanding of chemical reactions at the most fundamental level, which can explain reactive collisional processes with quantum state resolution and provide crucial information about reaction mechanisms, has been a significant and fundamental goal within modern physical chemistry.

The Born–Oppenheimer (BO) approximation is a fundamental concept in quantum chemistry that enables the solution of the Schrödinger equation for electrons at fixed nuclear configurations [1]. By assuming that the motion of nuclei is much slower than that of electrons, the BO approximation allows us to separate the electronic and nuclear degrees of freedom, giving rise to the concept of potential energy surface (PES). The PES represents the variation in potential energy as a function of nuclear coordinates, providing valuable information about the stability and energetics of chemical reactions. Depending on the size of the system and the desired level of accuracy, one can further investigate the nuclear motion of the system with an available PES either quantum mechanically or semi-classically [2,3] or classically. Classical simulations involve treating nuclei as classical particles obeying Newton's laws of motion. In contrast, quantum mechanical simulations consider the wave-like behavior of nuclei, often described using techniques such as wave packet dynamics or molecular dynamics based on quantum mechanics [4–9]. Even when the BO approximation is no longer valid, PESs remain powerful in reaction dynamics. In such cases, nonadiabatic couplings between different electronic states come into play as off-diagonal elements in the quasi-diabatic Hamiltonian, where the derivative couplings are minimized [10].

Consequently, constructing an accurate PES is of central importance for reaction dynamics calculations, which serves as a prerequisite for an accurate understanding of the most detailed product information and the underlying mechanisms in chemical reactions. Generating a highly accurate analytical representation of discrete high-level *ab initio* data spanning a wide configuration space is essential to achieve an accurate PES. Owing to advances in computational capacity, gold standard coupled cluster calculations [11], as well as multi-reference methods, such as MRCI [12], are suitable for determining the accurate potential energies for many systems. It is even more challenging to develop and implement the most accurate and efficient approach for analytically representing *ab initio* data, particularly for an increased number of degrees of freedom when the system becomes larger.

Decades ago, some linear or nonlinear fitting and interpolation approaches were employed to represent *ab initio* energies [13,14]. Due to their relatively simple mathematical form and the small *ab initio* dataset encompassing only a few degrees of freedom, these approaches can be highly efficient and accurate for small systems with no more than four atoms. Nevertheless, when extended to larger systems, the traditional approaches encountered limitations in evaluation efficiency or accuracy, reaching a bottleneck. The introduction of linear least squares fitting using permutationally invariant polynomials (PIPs) by Braams and Bowman has significantly propelled the advancement of PESs for polyatomic reactions [15]. This approach has been successful due to the enforcement of the permutational symmetry and proposal of primary and secondary invariants as the basis for fitting [16–20]. The PIP method has been successfully applied to construct accurate PESs for many polyatomic systems. One recent example is a 10-atom OH + C_2_H_6_ reaction accomplished by Czakó and coworkers [21], as well as many other reactions in the gas phase [20–24]. Bowman and coworkers applied the PIP method to many nonreactive systems with up to 15 atoms, such as tropolone [25,26], *N*-methyl acetamide [27], and acetyl acetone [28,29]. PESs with PIPs are actively being developed and used effectively in dynamics calculations. In addition, gradient evaluations via PIP PESs have been dramatically accelerated by using the reverse differentiation approach. A comprehensive evaluation of the training and testing precision of PIPs against numerous machine-learning methodologies was conducted using the MD17 database [30], showing that the PIP fits are as precise as those using machine-learning methods and the PIP method is computationally faster. PIPs are also used in Gaussian process regression to represent high-dimensional PESs [31].

In recent years, this field has witnessed the emergence of machine learning based approaches as efficient and precise methods for representing potential energies [32,33]. In particular, numerous efforts have been made to construct highly accurate multidimensional PESs based on artificial neural networks (NNs) [34–47]. Accurate PESs for small gas-phase systems were constructed using NN fitting with pairwise internuclear distances as inputs [48,49]. However, these NN PESs fail to enforce the crucial permutational symmetry of identical atoms, a fundamental property required for molecular dynamics simulations. To address this limitation, Jiang and Guo proposed using PIPs as descriptors for representing NN potentials with permutational symmetry [50]. This approach, known as the PIP–NN method, employs PIPs as inputs for NN fitting, enabling accurate representation of PESs for molecular systems containing up to seven atoms [51]. It was found that the plain NN approach, by averaging over the permutations, worked just as well as the PIP–NN approach when the spectrum of CH_4_ was calculated [52]. However, the NN approach without inherent permutational symmetry can induce additional errors in dynamics simulations for reactive systems when information on force is needed. A reported example is the H + CH_4_ reaction, for which the exchange scheme by averaging over permutations was used to approximately adapt the permutation symmetry. However, the computed forces have problems at high symmetry configurations, and the evaluation of the PES is very slow due to the smoothing strategy [53].

As the molecular size and the number of identical atoms increase, the efficiency of the PIP–NN method becomes considerably limited to small systems due to the redundancy of PIPs. Although PIPs can be truncated to a certain degree in practical applications, the basis remains incomplete, leading to additional difficulties in yielding PESs with small fitting errors.

Recently, our research group proposed a more efficient approach using fundamental invariants (FIs) as the input vector for constructing PESs with permutational symmetry [54,55]. The FIs are a new set of PIPs containing the least number of invariants, which can generate all the invariant polynomials. This method, referred to as FI-NN, can construct any permutation invariant PES for reactive systems with more than 10 atoms [56]. In contrast to the monomial symmetrization [57] in the PIP–NN method [50], FIs minimize the number of invariant polynomials while ensuring mathematical completeness and precision. Consequently, FI-NN significantly reduces the number of input invariants and fitting/evaluation times of PESs, which can be further extended to construct accurate PESs for larger systems with more atoms. The recent progress made for accurate FI-NN PESs includes gas-phase reactions with less than 10 atoms [58–68] and with up to 15 atoms [56], gas–surface reactions on rigid surfaces [69–73], and accurate representation of diabatic PESs [74–76].

This paper will be a short review of important research progress on accurate FI-NN PESs developed by this group and novel reaction mechanisms elucidated by dynamics simulations based on accurate global full-dimensional PESs. In the next sections, we will briefly discuss the FI-NN fitting methods and provide detailed illustrations of PESs and dynamics for gas-phase reactions relevant to combustion, atmospheric, and organic chemistry. Finally, we conclude with a brief summary of this review.

## FI-NN APPROACH

### Fundamental invariants

The computation of FIs for a given system can be accomplished using computational algebra software such as Singular [77]. However, this ‘black box’ approach is constrained by significant computational costs, particularly as the molecular size increases. As a result, the FIs generated by Singular are currently limited to systems with less than five atoms [54]. Thus, developing an efficient and user-controlled methodology for generating FI polynomials applicable to larger systems becomes crucial. Such an approach would enable the practical implementation of the FI-NN method for systems with more than five atoms, expanding its feasibility and applicability [55].

Because a comprehensive explanation of our approach to generating FIs has been previously presented [55], we will only provide a brief overview here. Our method uses King's algorithm [78] and computational invariant theory [79]. In King's algorithm, the FIs are directly obtained without differentiating primary and irreducible secondary invariants.

In the following we illustrate how the FIs are calculated by taking the prototypical four-atom A_2_B_2_ as an example. We assume field *K* is algebraically closed, and *G* is a finite group linearly acting on a polynomial ring *R* over the field. For a given system namely A_*i*_B_*j*_…X_*p*_, *G* = *S_i_* × *S_j_*×…×*S_p_*, which is the direct product of each symmetry group. For instance, for the A_2_B_2_ system, *G* = *S*_2_ × *S*_2_, which correspond to:


\begin{eqnarray*}
{{G}}( {{{\mathrm{A}}}_2{{\mathrm{B}}}_2} ) = \left[ {\begin{array}{@{}*{4}{c}@{}} 1\,\,&\,\,2\,\,&\,\,3\,\,&\,\,4\\ 1\,\,&\,\,2\,\,&\,\,4\,\,&\,\,3\\ 2\,\,&\,\,1\,\,&\,\,3\,\,&\,\,4\\ 2\,\,&\,\,1\,\,&\,\,4\,\,&\,\,3 \end{array}} \right].
\end{eqnarray*}

The invariant ring *R^G^* = {*r* ∈ *R*: *g*(*r*) = *r*, ∀*g* ∈ *G*} is a finitely generated subalgebra of *R*. An invariant ring Rey(*r*) called Reynolds operator can be obtained as the minimal set of homogeneous generators for invariant ring *R^G^*, based on the King's algorithm. Rey(*r*) is also a subring of *R* with all elements invariant under the permutation operator *ĝ*. For the A_2_B_2_ system, the following expressions are satisfied according to the labels of internuclear distances:


\begin{eqnarray*}
\!\!\!\!\!\!{{\mathrm{A}}}_2{{\mathrm{B}}}_2\!:\left( {{{{r}}}_2,\ {{{r}}}_{3},\ {r}_4,\ {{{r}}}_5} \right)\ \equiv \ \left( {{{{r}}}_{13},\ {{{r}}}_{14},\ {{{r}}}_{23}{{,}}\,{{{r}}}_{24}} \right).
\end{eqnarray*}

The Reynolds operators for the three systems (Rey(*r*)) can be further obtained below:


\begin{eqnarray*}
{\mathrm{Rey}}( {{{\mathrm{A}}}_2{{\mathrm{B}}}_2} ) = \left[ {\begin{array}{@{}*{4}{c}@{}} 2\,\,&\,\,3\,\,&\,\,4\,\,&\,\,5\\ 3\,\,&\,\,2\,\,&\,\,5\,\,&\,\,4\\ 4\,\,&\,\,5\,\,&\,\,2\,\,&\,\,3\\ 5\,\,&\,\,4\,\,&\,\,3\,\,&\,\,2 \end{array}} \right].
\end{eqnarray*}

The matrix we are referring to contains all symmetric internuclear distances (where ‘*r_i_*’ is simplified to ‘*i*’) in each row, and it plays a pivotal role in our algorithm. We designate the dimension of each row as ‘n_r_’ and the dimension of each column as ‘O_F_’, which corresponds to both the symmetric number and the size of each column in the matrix *G*. Here O_F_ is calculated as


\begin{eqnarray*}
{{\mathrm{O}}}_{\mathrm{F}}( {{{\mathrm{A}}}_2{{\mathrm{B}}}_2}) = 2! \times 2! = 4.
\end{eqnarray*}

The variable d(*R^G^*) is characterized as the maximum degree observed in fundamental invariants for *R^G^*. As per Noether's bound, it is established that d(*R^G^*)≤ |*G*|. However, the resulting invariant rings can prove to be surprisingly extensive, even when |*G*| is not notably large. To tackle this, a beneficial approach is to calculate the homogeneous Gröbner basis incrementally, exploiting the fact that the generators of the invariant ring are already known in ascending degrees. Furthermore, the partially computed Gröbner basis can serve in identifying new generators within the invariant ring.

In practice, for an *n*-atom molecular system with *m* identical atoms, the Rey(*r*) matrix size is *n*(*n* − 1)/2 × *m*!. Consequently, traversing the entire matrix becomes computationally expensive as the number of atoms increases. This process has been identified as the rate-limiting step in the computational procedure. To address this issue, we have incorporated OpenMP and MPI parallel processing into our program, resulting in accelerated evaluation of the Gröbner basis. Notably, Houston and coworkers recently reported on the latest software to fit PESs with PIPs [80].

Additionally, when the number of degrees exceeded one, we exploited the linear independence to directly eliminate redundant invariants, regardless of whether they are primary or secondary invariants within the current FI approach. The same procedure was applied to discard redundant polynomials for higher degrees.

Consider a vector space *V* defined over the field *K*, let {*v_i_* | *i* ∈ *I*} represent a set of elements in *V*, which are generated from invariant polynomials (*p*_1_, *p*_2_,…, *p_n_*). The group is linearly dependent over *K* if there exists a finite collection {*a_j_* | *j* ∈ *J*} of non-zero elements from *K*, such that


\begin{eqnarray*}
\mathop \sum \limits_{{j{\in}J}} {{{a}}}_{{j}}{{{p}}}_{{j}}\,{\mathrm{ = 0}}{\mathrm{.}}
\end{eqnarray*}

Here, *n_x_* denotes the number of randomly generated instances, and we denote the FI polynomials as *A* = (*p*_1_, *p*_2_, …, *p_n_*). Now, given a new polynomial *B* = *p_i_*, we express its linear independence using a loss function *L*(*p*):


\begin{eqnarray*}
{{L}}\left( {{p}} \right){\mathrm{ = \ }}\sqrt {\frac{{{\mathrm{min(}}{{\left( {{{A \times C - B}}} \right)}}^{\mathrm{2}}{{)}}}}{{{{{n}}}_{{x}}}}} .
\end{eqnarray*}

In this context, *C* represents the coefficient matrix, which can be generated using the linear least-squares method. When the value of *L*(*p*) is extremely small (close to 0), it indicates that the new polynomial *B* is redundant and should be removed.

Table 1 presents the FIs for selected molecules with more than five atoms. In the case of A_4_B_4_C_2_ and A_3_B_6_(A_2_B_7_), the results are truncated at degrees 5 and 6, respectively. This truncation is justified as FIs up to degree 5 or 6 provide adequate accuracy for use in the fitting process. For A_4_B_4_C_2_, there are 1546 FIs to degree 5, but this number is still too large to be used as the NN input. It therefore becomes necessary to implement a truncation process, limiting the selection to a more manageable subset, such as a range of 500–800 FIs, all within 5 degrees of complexity. Notably, this truncation approach was employed in our investigation of a 15-atom F^−^ + (CH_3_)_3_CI reaction [56]. This strategy, which decreases the number of FIs to a more reasonable range, has been validated to yield both efficiency and precision in FI-NN PESs.

**Table 1.** Number of FIs truncated by degree 7 for different systems.

System	Degrees 1–3	Degree 4	Degree 5	Degree 6	Degree 7
AB_5_	16	22	42	60	62
A_2_B_4_	22	26	37	32	5
A_2_B_5_	24	42	104	226	390
A_4_BCD	49	65	55	4	0
A_2_B_6_	25	48	147	449	1252
A_4_B_4_	39	79	236	687	1738
A_2_B_7_	25	50	166	586	–
A_3_B_6_	36	76	268	963	–
A_4_B_4_C_2_	99	294	1153	–	–

As a result, the FI approach effectively reduces the number of PIPs. The strategy developed in our program enables the efficient generation of FIs for larger molecular systems. Given the considerable reduction in invariant polynomials, combining FI polynomials with robust and efficient NN technology, called the FI-NN approach, is advantageous for fitting high-dimensional PESs with permutational invariant symmetry. Furthermore, the choice of polynomial forms is not unique in terms of practical fitting. For instance, FIs incorporating Morse-like variables, such as *y_i_* = exp(−*r_i/_a*) or the inverse of internuclear distances *y_i_* = 1/*r_i_*, often yield superior performance. Our proposed FI-NN approach provides opportunities to construct accurate PI PESs for reactive systems with more than 10 atoms.

### NN with FI inputs

**Figure 1.**
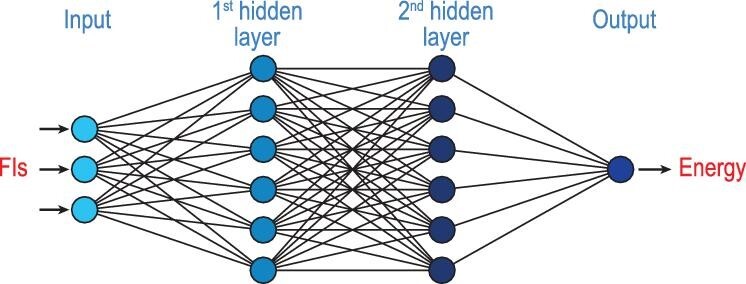
Illustration of the functional structure of a feed-forward neural network with two hidden layers for fitting PESs with FIs in the input layer.

NNs are versatile fitting methods capable of accurately fitting any analytical function. In our approach, we employed a feedforward NN with two hidden layers, connecting the input and output layers, as shown in Fig. 1. This architecture is denoted as I-J-K-1 NN, where I represents the number of inputs, corresponding to FIs in terms of polynomials of the internuclear distances of the configuration discussed above. The output layer provides the potential energy of the given configuration. The first hidden layer consists of *J* neurons, while the second hidden layer consists of *K* neurons. The input and output of the *j*^th^ neuron in the first hidden layer are given by


(1) \begin{eqnarray*}
x_j^1 = b_j^1 + \mathop \sum \limits_{i = 1}^I \big( {w_{ji}^1 \times {x}_i} \big),\ j = 1,2, \ldots ,J
\end{eqnarray*}

and


(2) \begin{eqnarray*}
y_j^1 = {f}^1\big( {x_j^1} \big),\ j = 1,2, \ldots ,J.
\end{eqnarray*}

The input and output of the *k*^th^ neuron in the second hidden layer are given by


(3) \begin{eqnarray*}
x_k^2 = b_k^2 + \mathop \sum \limits_{j = 1}^J \big( {w_{kj}^2 \times y_j^1} \big),\ k = 1,2, \ldots ,K
\end{eqnarray*}

and


(4) \begin{eqnarray*}
y_k^2 = {f}^2( {x_k^2}),\ k = 1,2, \ldots ,K.
\end{eqnarray*}

The output layer of NN can be expressed as follows:


(5) \begin{eqnarray*}
y_l^3 = b_l^3 + \mathop \sum \limits_{k = 1}^K \left( {w_{lk}^3 \times y_k^2} \right),\ l = 1,2, \ldots ,L,
\end{eqnarray*}

where *x_i_*(*i* = 1,*…, I*) denotes the input FIs. The *i*^th^ and *j*^th^ neurons of (*l −* 1)^th^ and *l*^th^ layers, respectively, are connected by weights $w_{j,i}^l$. The threshold of the *j*^th^ neuron of the *l*^th^ layer is determined by biases $b_j^l$. The transfer functions *f* ^1^ and *f* ^2^ are chosen as hyperbolic tangent functions. Throughout the NN fitting procedure, multiple tests can be conducted using different numbers of neurons for the two hidden layers based on a specific set of data points. This process aims to minimize the fitting error, as the NN structure considerably affects the quality of the fit. Once the specific NN structure is determined, the weights and biases in Eqs. (1)–(5) can be updated through appropriate NN training using the Levenberg–Marquardt algorithm [81]. The root mean square error (RMSE) is given by


(6) \begin{eqnarray*}
{\mathrm{RMSE}} = \sqrt {\frac{1}{n}\mathop \sum \limits_{i = 1}^n {{\left( {{E}_{fit} - {E}_{ab\ \scriptsize\textit{initio}}} \right)}}^2} ,\ \
\end{eqnarray*}

which is used to determine the fitting error. Furthermore, we used the ‘early stopping’ method to avoid overfitting. The entire dataset was divided into training and validation sets, and the training procedure was inhibited when overfitting occurred. We should have sufficient data points covering all the important regions on a global PES. Thus, a good sampling scheme for these data points is very important. If a molecular configuration from a trajectory is located far from the existing dataset, or the fitted energies for this configuration on several fittings are considerably different, we perform *ab initio* calculations for this data configuration and add it to the dataset for further NN fittings. We need to repeat the above iterative procedures many times until the PES is converged in terms of the number of data points.

## FI-NN PES

### H + C_2_H_2_ reaction

The H + HCCH → C_2_H_3_/H_2_ + C_2_H reaction, or its reverse, plays a vital role in combustion and interstellar chemistry. Extensive research has confirmed that the collision between HCCH and H leads to two distinct channels.


(R1) \begin{eqnarray*}
{\mathrm{H}} + {\mathrm{HCCH}} \leftrightarrow {{\mathrm{C}}}_2{{\mathrm{H}}}_3 \leftrightarrow {\mathrm{H}} + {{\mathrm{H}}}_2{\mathrm{CC}}
\end{eqnarray*}

(R2) \begin{eqnarray*}
{\mathrm{H}} + {\mathrm{HCCH}} \to {{\mathrm{H}}}_2 + {{\mathrm{C}}}_2{\mathrm{H}}
\end{eqnarray*}

However, due to the complex nature of the global PES for the H + HCCH reaction, there have been limited theoretical investigations into the reaction dynamics. Bowman and coworkers developed the first full-dimensional PES for the vinyl radical using PIP fitting [82]. The PIP PES describes the H + HCCH channel well but does not account for the H_2_ + C_2_H channel and high-energy H + H_2_CC. This is understandable because those regions were beyond the study scope at that time. Notably, a global PES for the larger allyl radical has been fitted by using the PIP method, which is quite complex and contains several product channels, including the high-energy isomer of acetylene (vinylidene) [83]. Yang *et al.* reported an NN PES for the reverse H_2_ + C_2_H → H + C_2_H_2_ reaction [84], but it does not cover the vinyl radical or the vinylidene (CH_2_C) + H channel. Consequently, these two local PESs cannot be employed for comprehensive dynamics calculations for the H + HCCH reaction.

It is essential to have an accurate global PES to accurately study the dynamics of this reaction. We recently developed a new global full-dimensional PES using FI-NN fitting based on ∼116 000 UCCSD(T)-F12a/aug-cc-pVTZ energies [61]. These UCCSD(T) energies were all calculated using the Molpro program [85]. For the C_2_H_3_ molecular system, there are 26 FIs with a maximum degree of 6, significantly smaller than the corresponding PIPs. Before calculating the FIs, the set of internuclear distances (*r_i_, i* = 1, 2, …, 10) was further displaced by Morse-like variables in all internuclear distances [*y_i_* = exp(−*r_i_*/$\lambda$), where $\lambda$ = 2.0]. Additionally, these 26 FIs were transformed to ${x}_i = p_i^{1/m}$, where *m* is the degree of the corresponding FI. Geometries for the *ab initio* database were selected properly from prior databases [82,84], and QCT calculations were performed using the preliminary and updated PESs.

**Figure 2.**
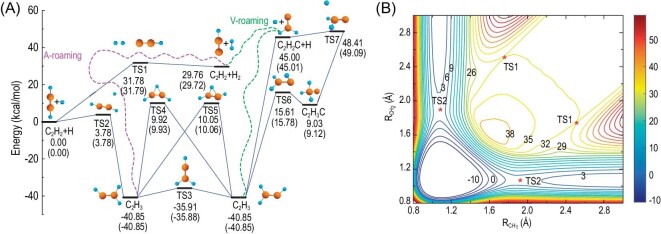
(A) Schematic of the FI-NN PES of C_2_H_3_, with stationary points and corresponding energies relative to reactants from the fitted PES and UCCSD(T)-F12a/AVTZ energies (depicted within parentheses). Dashed lines outline a sketch of the two roaming pathways (A-roaming and V-roaming). (B) Contour plot of the FI-NN PES as a function of two CH internuclear distances (R_CH_1__ and R_CH_2__) of C_2_H_3_, with other degrees of freedom optimized. (Revised from Ref. 61).

This fitting procedure resulted in an overall RMSE of only 9.84 meV for energies up to 6.0 eV relative to the global minimum of the PES. Fig. 2(A) illustrates all relevant stationary-point structures and energies on the FI-NN PES of the H + C_2_H_2_ reaction. The FI-NN PES accurately represents all reaction pathways, including the vinylidene channel and the isomerization pathways of vinyl and methylcarbyne. Fig. 2(B) shows the contour plot of the FI-NN PES, indicating its dependence on two C–H internuclear distances of C_2_H_3_ (R_CH_1__ and R_CH_2__), with the remaining coordinates (degrees of freedom) fully optimized. This contour plot effectively portrays the energies and positions of various species involved in the reaction: reactant species, product species, transition states, and intermediate complexes. Within this plot, we can observe two distinct reaction pathways. The first pathway involves H atom addition and elimination via transition state 1 (TS1) and the C_2_H_3_ complex, leading to the sequential transformation of H + C_2_H_2_ → C_2_H_3_ → C_2_H_2_ + H. The second pathway is characterized by H atom abstraction from C_2_H_2_, resulting in the formation of H_2_ and C_2_H via TS1. Overall, these reaction pathways demonstrate remarkable smoothness and coherence.

Owing to the accuracy of global FI-NN PES, dynamics simulations based on this PES revealed intricate reaction mechanisms [61]. Apart from the conventional TS pathway involving direct abstraction, two intriguing and distinct roaming pathways have been proposed for the H + C_2_H_2_ → H_2_ + C_2_H reaction. These are acetylene-facilitated roaming (A-roaming) and vinylidene-facilitated roaming (V-roaming), as shown in Fig. 2(A). These double-roaming pathways dominate the reactivity at collision energies above 50 kcal/mol. Importantly, these three reaction pathways produce products with distinct energy partitions and angular distributions. Further dynamics simulations for the isotope reaction of C_2_D_2_ + H based on the FI-NN PES also elucidated interesting double-roaming pathways [86].

### H + C_2_H_4_ reaction

The H + C_2_H_4_*→* C_2_H_5_/H_2_ + C_2_H_3_ reaction plays a considerable role in combustion chemistry. However, due to the lack of a comprehensive global full-dimensional PES, the detailed dynamics and reaction mechanism of this crucial reaction have remained unclear for an extended period. We recently reported an accurate full-dimensional PES for the H + C_2_H_4_ reaction [62], encompassing H + C_2_H_4_*→* C_2_H_5_ and the conventional direct abstraction pathway leading to H_2_ + C_2_H_3_ products. The construction of the global PES involved FI-NN fitting to data obtained from ∼100 000 UCCSD(T)-F12a/aug-cc-pVTZ energies.

Starting from the first PES based on the initial 50 000 UCCSD(T)-F12a/aug-cc-pVTZ energies, more energy points were added interactively by performing QCT calculations on those preliminary PESs. Data points with significant energy deviations and errors were collected in each cycle. To avoid introducing redundant data points, we defined a criterion to measure the ‘distance’ between two geometries, denoted as *a* and *b*, using the following formula:


\begin{eqnarray*}
{{D}} = \left( {{{{E}}}_{{a}} - {{{E}}}_{{b}}} \right)\sqrt {\mathop \sum \limits_{i = 1}^N {{\left( {{r}_{a,i} - {r}_{b,i}} \right)}}^2} ,
\end{eqnarray*}

where *r_i_* (*i* = 1, …, *n*) represents the bond length of the *i*^th^ geometry. Data points that exhibited similarity in energy and geometry domains to existing data were discarded. Extensive QCT calculations of the H + C_2_H_4_ reaction were carried out to validate the convergence of the fitted PES with respect to the number of energy points. The final FI-NN PES, which is the average of five PESs with minimal RMSEs, results in an RMSE of 13.0 meV for energies up to 6.0 eV relative to the energy of H + C_2_H_4_, highlighting the extremely high precision for PESs governing seven-atom multichannel reactions.

**Figure 3.**
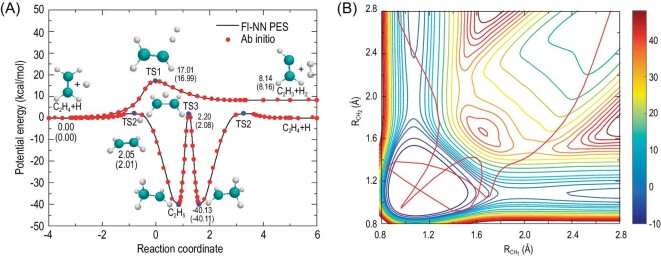
(A) Minimum energy paths of the H + C_2_H_4_ reaction obtained from the PES (solid curves) and the UCCSD(T)-F12a/AVTZ calculations (symbols). (B) Contour plot of PES as a function of two CH internuclear distances (R_CH_1__ and R_CH_2__) of C_2_H_5_, with degrees of freedom optimized. The evolution of one typical collision-induced roaming trajectory is superimposed on the contour plot with an extremely short reaction time. (Revised from Ref. 62).

Fig. 3(A) illustrates relevant stationary-point structures and energies derived from the PES and direct *ab initio* calculations relative to the H + C_2_H_4_ reagents (excluding vibrational zero-point energies) corresponding to the identified product channels. Determining the minimum energy paths involved the quadratic steepest descent method and demonstrated excellent agreement between the PES and UCCSD(T)-F12a/aug-cc-pVTZ results.

The dynamics calculations based on the full-dimensional FI-NN PES revealed a novel collision-induced roaming pathway to H_2_ + C_2_H_3_ and the complex-mediated roaming mechanism. This pathway, facilitated by bimolecular collisions, deviates from the conventional direct abstraction pathway and originates from an extremely short-lived intermediate. The collision-induced and complex-mediated roaming mechanisms show distinct angular distributions. However, both result in highly internally excited C_2_H_3_. One notable feature of the roaming mechanism is the production of highly internally excited C_2_H_3_, which offers potential for experimental observation. The FI-NN PES was also used to investigate the energy transfer dynamics of the H + C_2_H_4_ reaction [87].

The two-dimensional contour plot of the PES as a function of the two CH bonds (R_CH_1__ and R_CH_2__) is shown in Fig. 3(B), upon optimizing the remaining degrees of freedom. Moreover, we superimposed the trajectory of a representative roaming pathway onto the contour plot of the PES. This particular roaming pathway emerges due to collision-induced roaming dynamics and demonstrates an extraordinarily rapid reaction time. The roaming trajectories can only occur at very high collision energies, as evidenced by a barrier of ∼30 kcal/mol from C_2_H_5_ to H_2_ + C_2_H_3_. This observation highlights that roaming pathways are exclusively accessible under specific energy conditions.

### CH_2_OO + H_2_O reaction

It is widely recognized that the ozonolysis of alkenes in the troposphere proceeds through the involvement of Criegee intermediates (CIs). Considering the abundant presence of water vapor, the reaction with water molecules is considered the dominant pathway in the atmosphere. Consequently, extensive research efforts have been made to investigate the reaction of CIs with water [88,89]. Despite the predominant reaction with water dimer, the ultimate products resulting from the interaction between the simplest CI (CH_2_OO) and water monomer remain controversial in both experimental and theoretical studies.

To gain insights into the reaction mechanism of CH_2_OO + H_2_O and elucidate the associated end products, we developed the first full-dimensional PES for the CH_2_OO + H_2_O system using FI-NN fitting to a large dataset comprising UCCSD(T)/aug-cc-pVTZ data points [65]. Considering the vast configuration space encompassed by the investigated system, we divided the dataset into two distinct regions: the reactant asymptotic region and the interaction region. To overcome the significant challenges associated with simultaneously fitting all the data points, we allowed these two regions to overlap within a switching zone. Notably, atomic permutative symmetries between H_2_O and CH_2_OO do not need to be considered in the reactant asymptotic region. The number of FIs in the asymptotic region is 143. However, this number increases to 505 for the interaction region, although truncated to fifth-order terms. Ultimately, the two parts were connected using a smooth switch function, ensuring a seamless transition between them. Our initial PES was constructed based on an initial dataset, and additional data points were iteratively added by performing QCT calculations on the preliminary PES. A total of 111 076 energy points were finally included in the fitting base, resulting in a final RMSE of 15.5 meV.

**Figure 4.**
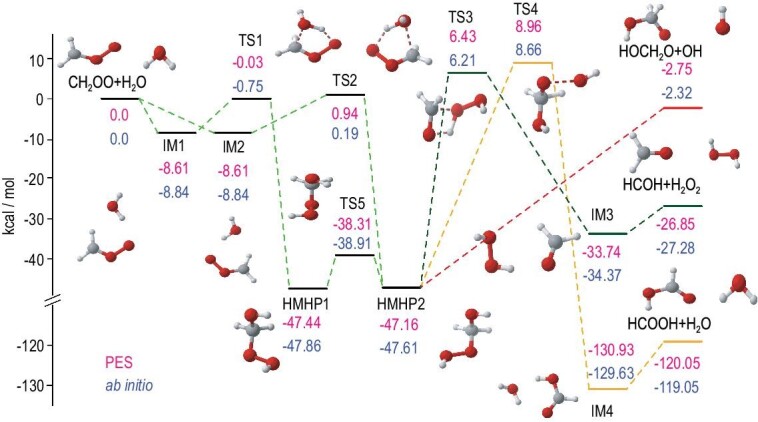
PES diagram of the CH_2_OO + H_2_O reaction, with all energy values presented in kcal/mol relative to the CH_2_OO + H_2_O asymptote at different levels: FI-NN PES (indicated in red) and CCSD(T)-F12a/AVTZ (indicated in blue). (Adapted from Ref. 65).

Fig. 4 depicts the energy schematics and configurations of stationary points with different pathways and product channels for the CH_2_OO + H_2_O reaction. A comparison between the CCSD(T)-F12a/aug-cc-pVTZ and PES energies for all stationary points associated with different pathways demonstrates good agreement between the two result sets. The resulting full-dimensional PES includes the regions from reactants to hydroxymethyl hydroperoxide (HMHP) intermediates and covers different end-product channels. Consequently, it has made reliable and efficient kinetics and dynamics calculations possible.

The total rate coefficient of CH_2_OO + H_2_O → HMHP was computed using the canonical variational transition state (CVT) theory with small curvature tunneling (SCT) corrections in combination with the PES interface. The computed rate coefficient agrees well with experimental results and previous theoretical findings. Extensive QCT calculations were performed to determine the converged product branching ratios. The barrierless HOCH_2_O + OH channel was found to play an important role. The highly exothermic channel HCOOH + H_2_O accounts for ∼6% of the overall yield, while a negligible fraction leads to CH_2_O + H_2_O_2_. Our study represents the first full-dimensional dynamics investigation based on a comprehensive PES for the reaction between the simplest CI and a water monomer, offering valuable insights into the end product channels and providing kinetics and dynamics information.

### F^−^(H_2_O) + CH_3_I reaction

Bimolecular nucleophilic substitution (S_N_2) reactions have garnered significant attention in chemical reaction dynamics due to their fundamental significance in organic chemistry. Over the past few decades, extensive theoretical and experimental investigations have unveiled novel mechanisms elucidating the microscopic aspects of S_N_2 reactions [90, 91]. The participation of solvents has emerged as a crucial factor influencing the reactivity of these fundamental reactions.

The absence of analytical full-dimensional PESs for microsolvated reactions has limited dynamical simulations, which have predominantly relied on computationally expensive on-the-fly methods and have been confined to a few collision energies [92]. The development of such PESs has encountered considerable challenges, primarily due to the high dimensionality involved, as exemplified by the 21 degrees of freedom in the title monosolvated F^−^(H_2_O) + CH_3_I reaction.

We reported an accurate 21-dimensional PES for the F^−^(H_2_O) + CH_3_I reaction, enabling efficient and comprehensive dynamics investigations into this fundamental reaction, considering the influence of solvation [63]. While the FI method inherently minimizes the size of invariants compared to other invariant methods, the current system presents a significant number of FIs due to five identical hydrogen atoms. To address this problem, we considered hydrogen atoms in different groups as non-identical atoms during FI generation, as hydrogen atoms do not exchange between H_2_O and CH_3_. Thus, a concise molecule descriptor comprising 277 invariants was successfully created, significantly reducing the evaluation time for potential energy calculations. These 277 FIs were then input to a double hidden-layer NN.

Energies of ∼140 000 geometries were computed using CAM-XYG3, which combines the doubly hybrid density functional XYG3 [93] and long-range correction with the Coulomb-attenuating method (CAM) [94]. Practical implementation of the FCH_3_ + I^−^ + H_2_O region was challenging due to insufficient sampling in the vast spatial freedom associated with its three-body characteristics. The FCH_3_ + I^−^ + H_2_O region and other regions were segmented and fitted separately to improve the fitting. The global FI-NN PES was obtained by smoothly connecting the two fitted parts using weight functions.

For the FCH_3_ + I^−^ + H_2_O region, each energy was treated as the sum of the energies of the individual fragments and their interaction energies. Therefore, the PES function is described as follows:


\begin{eqnarray*}
{V}_{{\mathrm{FC}}{{\mathrm{H}}}_3 + {{\mathrm{I}}}^ - \!{\mathrm{\ }} +\! {\mathrm{\ }}{{\mathrm{H}}}_2{\mathrm{O}}}{\mathrm{\ }} = {\mathrm{\ }}{V}_{{\mathrm{FC}}{{\mathrm{H}}}_3} + {V}_{{{\mathrm{H}}}_2{\mathrm{O}}} + {V}_{\textit{interaction}}.
\end{eqnarray*}

The interaction PES was constructed using the FI-NN method by fitting the interaction energies of geometries in the dataset. The energy-splitting scheme was employed to address the issue of insufficient sampling in the three-body region, leveraging the small variation in the interaction PES. Only 30 000 energies were used to fit the three-body region, resulting in a small fitting error of 6 meV. In contrast, without employing the energy-splitting scheme, the fitting error increased to 16 meV. Similarly, a new PES for the unsolvated F^−^ + CH_3_I reaction was developed through FI-NN fitting using CCSD(T)/aug-cc-pVTZ (-PP for the iodine atom) data points. The resulting fitting of the monosolvated and unsolvated reactions yielded overall RMSEs of 10.0 meV and 4.5 meV, respectively.

**Figure 5.**
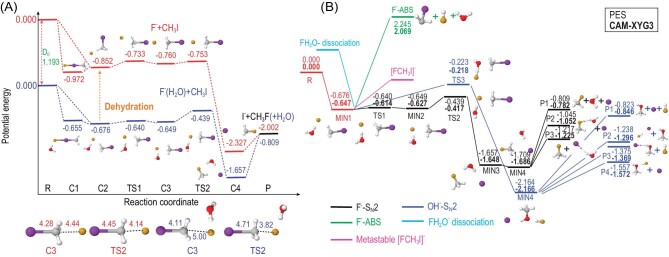
(A) Schematic potential profiles (in eV) for the S_N_2 reactions of F^−^ + CH_3_I → CH_3_F + I^−^ (red curve) and F^−^(H_2_O) + CH_3_I → CH_3_F + I^−^ + H_2_O (blue curve). The energy values are provided relative to the reactants in eV. In the bottom panel, the ion-dipole minima (C2) structures and the inversion transition state (TS2) are displayed, with the F–C and C–I bond lengths noted in Bohr units for reference. (B) Detailed potential energy profile of the F^−^(H_2_O) + CH_3_I reaction, showing the PES and CAM-XYG3 energies of stationary points on multiple pathways listed in the text. The energies are relative to the reactants in eV, and the stationary points are shown on the PES. (Revised from Ref. 63).

Fig. 5(A) shows the schematic representations of the PESs for F^−^ + CH_3_I → CH_3_F + I^−^ and F^−^(H_2_O) + CH_3_I → CH_3_F + I^−^ + H_2_O, displaying the relevant stationary points and energies along the minimum-energy paths, which offer insights into the underlying dynamics. Overall, the presence of a single water molecule does not considerably alter the topography of the PES. The PES features potential wells associated with the front side of the complex, hydrogen-bonded complex, and ion-dipole complex preceding the submerged inversion transition state, along with a deep minimum corresponding to the product complex after inversion. With the inclusion of hydrogen bonds between H_2_O and other species, the energetics and geometries of stationary points in the monosolvated reaction differ from those of the unsolvated reaction. A complete description of the global full-dimensional PES for F^−^(H_2_O) + CH_3_I, including the F-S_N_2 pathways leading to hydrated products and OH-S_N_2 pathways, is provided in Fig. 5(B).

We anticipate that the ability to construct a comprehensive PES for this monosolvated reaction will be a pathway to conducting more rigorous investigations involving reactions with multiple solvent molecules. Such endeavors will undoubtedly yield profound insights into the intricate dynamics in the solution phase. This advancement is promising for providing a deeper understanding of molecular interactions within solvent environments.

### F^−^ + C(CH_3_)_3_I reaction

The competition between base-induced elimination (E2) and S_N_2 pathways has been particularly interesting, especially for methyl-substituted alkyl halides, where the E2 pathway becomes dominant. To directly investigate these reactions, crossed-beam scattering experiments under single-collision conditions were developed by Wester *et al.*, allowing for the imaging of differential cross-sections (DCSs) for reactions between anions and methyl-substituted alkyl halides (X^−^ + RY) [95,96]. It was observed that S_N_2 reactivity decreased with increasing methyl substitution, such as in the cases of F^−^ + CH_3_I, F^−^ + C_2_H_5_I, F^−^ + ^i^C_3_H_7_I, and F^−^ + ^t^C_4_H_9_I. In particular, for F^−^ + ^t^C_4_H_9_I, the measured DCSs suggest that the E2 pathway dominates, while the contribution from S_N_2 is nearly negligible [95].

We recently developed an accurate full-dimensional (39-dimensional) PES for the F^−^ + ^t^C_4_H_9_I reaction involving 15 atoms [56], covering both E2 and S_N_2 pathways. This PES was constructed by FI-NN fitting to ∼220 000 energy points calculated using the CAM-XYG3/augcc-pVTZ (aug-cc-pVTZ-PP for iodine atom) method.

Considering the enormous configuration space of the investigated system, we employed space partitioning and energy-splitting methods to overcome the challenges associated with fitting all the data points. Specifically, we collected geometries using energy splitting for the asymptotic region of the reactants F^−^ + (CH_3_)_3_CI. An accurate local PES was constructed for tert-butyl iodine ((CH_3_)_3_CI) using the CAM-XYG3 method. To improve the accuracy and efficiency of the fitting process, we divided the configuration space into three partitions: (1) the asymptotic region of the reactants (with a distance between the center of mass of F^−^ and (CH_3_)_3_CI larger than 7 Å), (2) the interaction region, and (3) the asymptotic region of the E2 product channel (with a minimum distance between the center of mass of the three products (CH_3_)_2_C = CH_2_ + HF + I^−^ larger than 5.5 Å). Smooth connections between adjacent regions were achieved using a switch function. The energies of Part 3 were split into the energy of the three fragments and their interaction energies, following the same procedure as for Part 1. Additionally, accurate sub-PESs for (CH_3_)_2_C = CH_2_ and HF were constructed.

Although FIs reduce the number of invariants, the F^−^ + (CH_3_)_3_CI reaction still involves 1282 FIs with a maximum degree of three without considering the permutation symmetry of hydrogen atoms on different methyl groups. FIs were truncated at a maximum of 500 to manage the complexity, which has been demonstrated to be sufficient for obtaining an accurate fit. The NN structure 500–50–100–1 was selected to fit the energy points in the interaction region (Part 2), as well as the interaction energies between the reactant (Part 1) and product fragments (Part 3). In addition, 746 FIs up to degree 3 were utilized to construct the local PES for (CH_3_)_3_CI, employing 50 and 100 neurons in the first and second hidden layers, respectively. Furthermore, the (CH_3_)_2_C = CH_2_ PES was trained using the NN structure 606–10–100–1, incorporating all FIs up to degree 3. Overall, the total RMSE of all data points of the three parts is only 8.3 meV.

**Figure 6.**
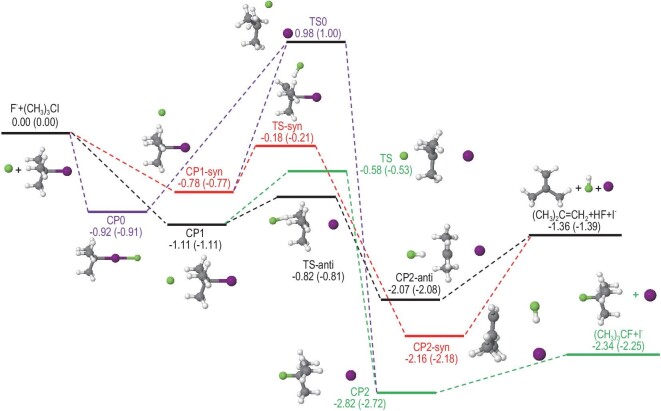
Schematic PES of the F^−^ + (CH_3_)_3_CI reaction including multiple pathways: backside attack S_N_2 (green curve), frontside attack S_N_2 (purple curve), anti-E2 (black curve), and syn-E2 (red curve). The relative energies obtained from CAM-XYG3/AVTZ(-PP) and CCSD(T)/AVTZ(-PP) (in parentheses) are in eV. (Adapted from Ref. 56).

**Figure 7.**
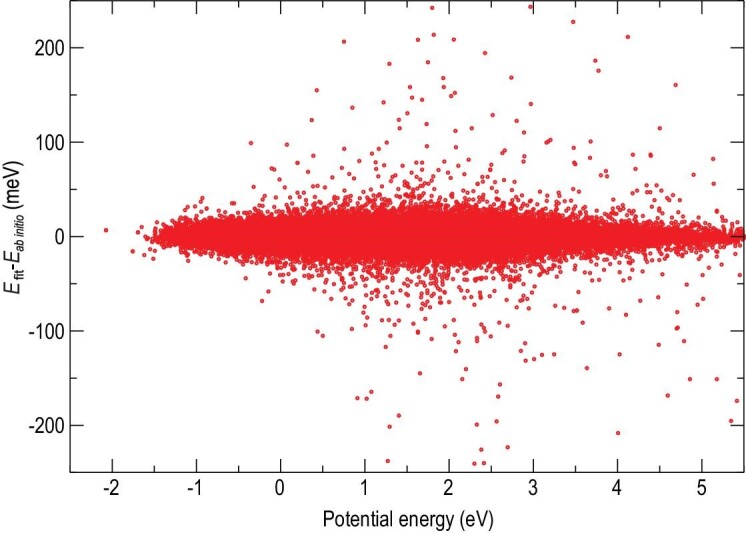
Fitting errors for data points on the FI-NN PES as a function of their corresponding CAM-XYG3/AVTZ(-PP) energies relative to the energy of the reagent. (Revised from Ref. 56).

**Figure 8.**
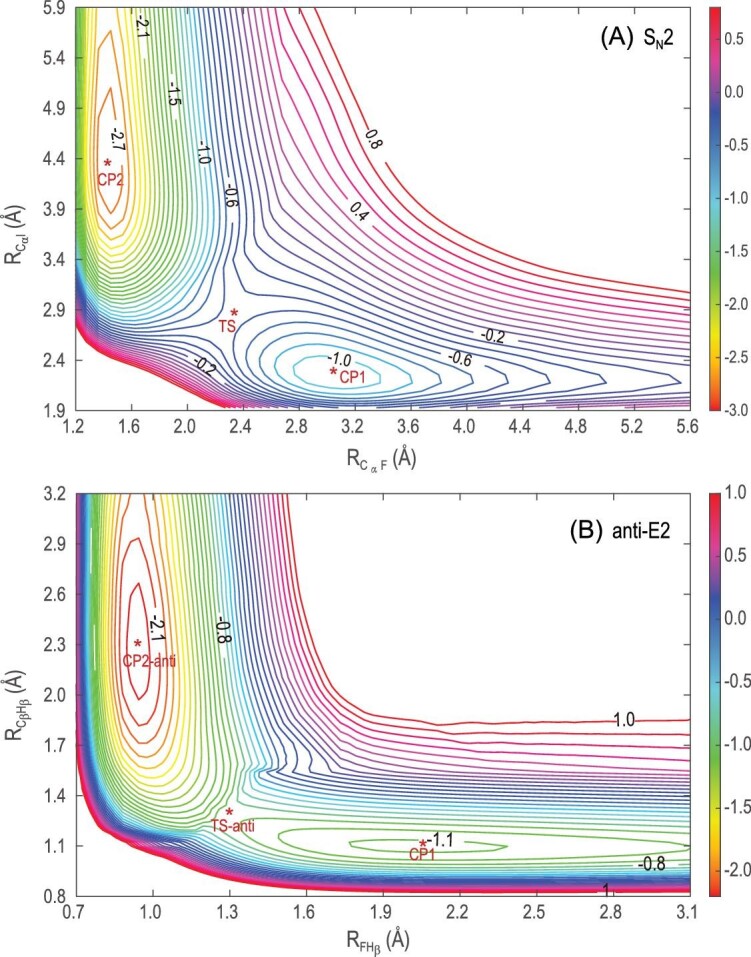
Two-dimensional contour plots of the backside attack S_N_2 (A) and anti-E2 (B) channels with the remaining degrees of freedom fully optimized on the FI-NN PES. (Adapted from Ref. 56).

As illustrated in Fig. 6, both the backside attack S_N_2 and E2 reactions are barrierless and highly exothermic. They involve pre-reaction wells on the reactant side and post-reaction wells on the product side. The distribution of fitting errors for all the data points shown in Fig. 7 represents the high accuracy of FI-NN fitting for this 39-dimensional reaction. To further validate the accuracy of the full-dimensional PES, we present two contour plots illustrating the backside attack S_N_2 and anti-E2 pathways in Fig. 8. These contour plots were generated using full-dimensional optimization based on the global full-dimensional PES. Notably, these contours exhibit smooth behavior, accurately representing the positions and energies of the reactants, pre-reaction minima, TSs, post-reaction minima, and products. This is the first report of contour plots for such large reactive systems. The exceptional quality of these contours emphasizes the high accuracy of the current fitting, the reliability of the 15-atom multi-channel PES, and the robustness of the FI-NN approach.

This comprehensive PES allows for efficient dynamics simulations. By employing extensive QCT simulations with the aid of this global PES, we not only reproduced the experimental measurements but also uncovered the true dynamic origin behind the reduced S_N_2 reactivity and increased E2 reactivity in the F^−^ + (CH_3_)_3_CI reaction. In contrast to the commonly observed steric hindrance effect on S_N_2 reactivity due to bulky substitution, our findings indicate that the suppression of S_N_2 is primarily attributed to the high reactivity of the three methyl groups, favoring the E2 pathway. Using the current analytical PES, we can confirm that in the absence of E2 competition, the ‘intrinsic’ reactivity of S_N_2 can still be very high, even in the presence of steric congestion. These results challenge the conventional notion of steric hindrance as the sole factor governing S_N_2 reactivity and highlight the importance of considering the specific reactive environment and competing reaction pathways in understanding and predicting reaction outcomes.

## CONCLUSIONS AND OUTLOOK

This review presents just a fraction of the reactive system for which the accurate and efficient FI-NN approach, together with high-level electronic structure calculations, has enabled global, full-dimensional PESs and detailed reaction dynamics studies, focusing particularly on multi-channel polyatomic reactions of importance in combustion, atmospheric, and organic chemistry. The PESs constructed by the FI-NN approach demonstrate an exceptional degree of complexity, molecular size, and fitting accuracy (∼meV), facilitating the identification of unrecognized and novel reaction pathways and the determination of intrinsic reactivity at a quantitively accurate level. Currently, the applicable size of the FI-NN approach has been pushed up to reactive systems with over 10 atoms. To our knowledge, the reported PES for the F^−^ + C(CH_3_)_3_I gas-phase reaction with 39 degrees of freedom represents the most extensive full-dimensional PES documented to date. Some FI-NN PESs are a linear combination of sub-PESs in different regions. While single fitting using FI-NN is not particularly challenging, applying this approach to a high-dimensional system, such as the 15-atom system F^−^ + C(CH_3_)_3_I, tends to yield larger fitting errors compared to fitting different regions of the PESs separately. Therefore, our approach involves segmented fitting to enhance fitting efficiency and accuracy.

Numerous significant reactions of comparable scale still lack comprehensive dynamical information, emphasizing the need for dedicated efforts to construct corresponding PESs and investigate the associated reaction dynamics. As we explore even larger systems, such as those exceeding 20 atoms, an extension of the FI-NN approach is highly anticipated. Notably, the computational efficiency of the FI-NN approach decreases for larger systems, particularly for those with over 30 atoms, due to the non-linear increase in FIs as molecules grow in size. This issue can be partially resolved by introducing segmented fitting in the reactant, interaction, and product regions using different symmetry groups with different degrees in FIs [56,63,65], which was inspired by the recent fragmented PIP approach [27]. In this context, the atomistic neural network (AtNN) technique, initially introduced by Behler and Parrinello [45], is a suitable point of comparison regarding accuracy. Alternative methods inspired by AtNN, including deep potential [97] and embedded atom neural network (EANN) [46], are viable options. Nevertheless, the fitting accuracy achieved by the FI-NN approach is exceptionally high. Consequently, there may be a trade-off with fitting accuracy when applying AtNN approaches to larger systems. Hence, it is crucial to establish the molecular size boundary where the FI-NN approach is not feasible and other methods become necessary. Furthermore, future endeavors exploring alternative descriptors will undoubtedly yield considerable advancements in the field.
